# GPATCH3, a splicing regulator that facilitates tumor immune evasion via the modulation of ATPase activity of DHX15

**DOI:** 10.3389/fimmu.2025.1612461

**Published:** 2025-08-11

**Authors:** Tingrong Ren, Gaigai Wei, Jingjing Yi, Yuqi Zhang, Haiping Zhao, Nana Wu, Huiling Zhang, Zhihan Guo, Yihan Wang, Jiating Kuang, Zhaoying Sheng, Duanwu Zhang

**Affiliations:** ^1^ Children’s Hospital of Fudan University, National Children’s Medical Center, and Shanghai Key Laboratory of Medical Epigenetics, International Co-laboratory of Medical Epigenetics and Metabolism, Ministry of Science and Technology, Institutes of Biomedical Sciences, Fudan University, Shanghai, China; ^2^ Institute of Pediatrics, Children’s Hospital of Fudan University, National Children’s Medical Center, Fudan University, Shanghai, China

**Keywords:** GPATCH3, DHX15, DEAH-box helicase, ATPase activity, splicing, alternative splicing, tumor microenvironment, immune evasion

## Abstract

**Introduction:**

Aberrant pre-mRNA splicing is increasingly recognized as a key contributor to tumorigenesis and immune evasion. However, the regulatory factors orchestrating splicing dynamics within the tumor microenvironment (TME) remain incompletely understood. Here, we identify GPATCH3, a previously uncharacterized G-patch domain–containing protein, as a critical modulator of alternative splicing and immune regulation in cancer.

**Methods:**

We employed biochemical studies, splicing reporter assays, and transcriptomic analyses to elucidate the function of GPATCH3. *In vitro* and *in vivo* models, including GPATCH3-depleted cell lines and mouse xenografts, were used to assess its roles in tumor progression. Immune infiltration patterns were analyzed using TIMER2.0 based on TCGA transcriptomic data.

**Results:**

GPATCH3 interacts with the RNA helicase DHX15 and enhances its ATPase activity, promoting proper spliceosome disassembly. Loss of GPATCH3 led to splicing alterations, including in immunoregulatory genes such as *CXCR3*, *CD44*, and *FOXP3*. Functional studies revealed that GPATCH3 deficiency attenuated tumor growth *in vivo*. Conversely, elevated GPATCH3 expression was associated with reduced infiltration of cytotoxic T cells and NK cells, alongside an enrichment of immunosuppressive populations such as MDSCs and CAFs across multiple cancer types. Transcriptomic analysis further revealed that GPATCH3 deficiency upregulates immunomodulatory genes such as *CXCL8* and *LAG3*, suggesting a role in shaping the TME via splicing regulation.

**Discussion:**

Our findings suggest GPATCH3 as a critical regulator that governs alternative splicing and immunosuppressive microenvironment remodeling. By modulating the splicing fidelity of key immune genes and altering their expression, GPATCH3 may facilitate immune escape and tumor progression. These results provide mechanistic insights into how RNA splicing factors interface with immune regulation and highlight GPATCH3 as a potential therapeutic target for immunomodulatory cancer therapy.

## 1 Introduction

The spliceosome, a highly dynamic and complex molecular machine, is essential for pre-mRNA processing (1). It comprises five major small nuclear ribonucleoproteins (snRNPs)—U1, U2, U4, U5, and U6—along with numerous associated splicing factors (1–3). During the splicing cycle, the spliceosome undergoes extensive structural remodeling to ensure precise intron excision and exon ligation (1–4). This intricate machinery is indispensable for generating transcriptomic diversity and maintaining normal cellular function (3, 4). However, dysregulation of core spliceosomal components or their regulatory cofactors can lead to aberrant splicing events, which are increasingly recognized as drivers of human disease, particularly cancer (5–9). For example, the splicing factor SRSF1 has been shown to promote tumor progression by regulating the production of pro-oncogenic isoforms (10, 11).

In recent years, the spliceosome has emerged as a promising therapeutic vulnerability in cancer (12, 13). Cancer cells exhibit heightened dependency on accurate splicing and are often hypersensitive to perturbations of the splicing machinery, offering a therapeutic window (14). One notable example is the SF3B1-targeting splicing modulator E7107, which has shown efficacy in preclinical models of leukemia and solid tumors (15–17). These findings highlight the potential of targeting spliceosomal components and their cofactors for cancer therapy.

GPATCH3 (G-Patch Domain Containing 3), a member of the G-patch protein family, has been implicated in antiviral immunity via suppression of RIG-I-like receptor signaling and type I interferon responses (18). G-patch proteins are characterized by a glycine-rich motif that enables interaction with DEAH-box helicases such as DHX15, modulating their ATPase and helicase activities to regulate RNA metabolism (19–22). This interaction is critical not only for pre-mRNA splicing, but also for ribosome biogenesis and RNA surveillance. Recent evidence links dysregulated G-patch proteins to multiple disease processes, including cancer (23–26).

DHX15, a DEAH-box RNA helicase, plays a pivotal role in spliceosome remodeling and RNA metabolism (27, 28). It cooperates with G-patch cofactors to facilitate RNA unwinding and spliceosomal transitions during catalysis, underscoring the importance of helicase–cofactor interplay in splicing fidelity (25).

In this study, we identify GPATCH3 as a previously unrecognized regulator of pre-mRNA splicing that functionally interacts with DHX15 to support its ATPase activity, thereby safeguarding normal alternative splicing. Nevertheless, overexpression of GPATCH3 also leads to pronounced alterations in splice site selection and splicing patterns. Notably, GPATCH3 is significantly upregulated across multiple cancer types and correlates with adverse patient prognosis. Mechanistically, we show that GPATCH3 reprograms the tumor immune microenvironment by limiting cytotoxic immune cell infiltration while increasing immunosuppressive populations—a phenotype potentially linked to its splicing regulatory activity. These findings unveil a critical role for GPATCH3 in modulating RNA splicing and tumor immunity and suggest that it may represent a tractable therapeutic target for spliceosome-based cancer interventions.

## 2 Results

### 2.1 GPATCH3 is associated with the spliceosome

**Figure 1:**
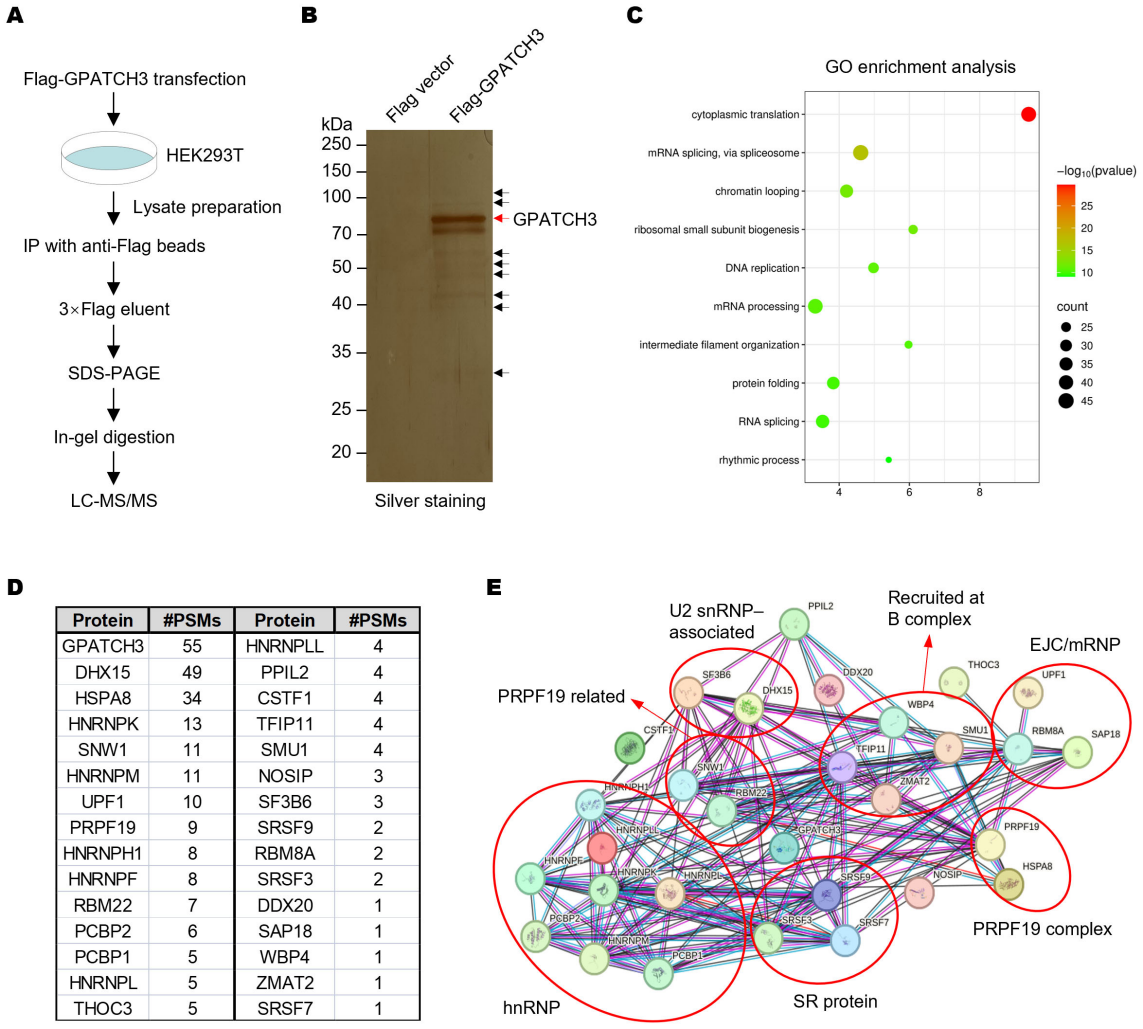
GPATCH3 associates with core spliceosomal complexes. **(A)** Schematic of the workflow for identifying GPATCH3-interacting proteins by co-immunoprecipitation followed by mass spectrometry (Co-IP/MS). **(B)** HEK293T cells were transfected with Flag vector or Flag-tagged GPATCH3. Cell lysates were immunoprecipitated using anti-Flag M2 magnetic beads, and bound proteins were visualized by silver staining. **(C)** Gene Ontology (GO) enrichment analysis of GPATCH3-interacting proteins identified by Co-IP/MS. **(D)** Splicing-related proteins enriched in the GPATCH3 immunoprecipitates. **(E)** Spliceosome regulatory network associated with GPATCH3 derived from the Co-IP/MS dataset. Network was constructed by STRING online software (https://cn.string-db.org/).

The G-patch protein family is defined by a conserved glycine-rich structural motif. Several members, including GPATCH1, GPATCH8, and GPATCH11, play critical roles in RNA splicing (21, 24, 29). However, the function of GPATCH3 remains poorly characterized. To explore whether GPATCH3 is involved in RNA splicing, we performed co-immunoprecipitation coupled with mass spectrometry (Co-IP/MS) to identify GPATCH3-interacting proteins (**Figures 1A, B**; **Supplementary Data Sheet S1**). Gene Ontology (GO) enrichment analysis revealed that the GPATCH3-associated proteins are significantly enriched in splicing-related biological processes (**Figure 1C**). Interaction network analysis further demonstrated that GPATCH3 is primarily associated with components of the U2 snRNP and the PRPF19 complex, both of which are core elements of the spliceosome (**Figures 1D, E**).

### 2.2 GPATCH3 physically interacts with DHX15

**Figure 2:**
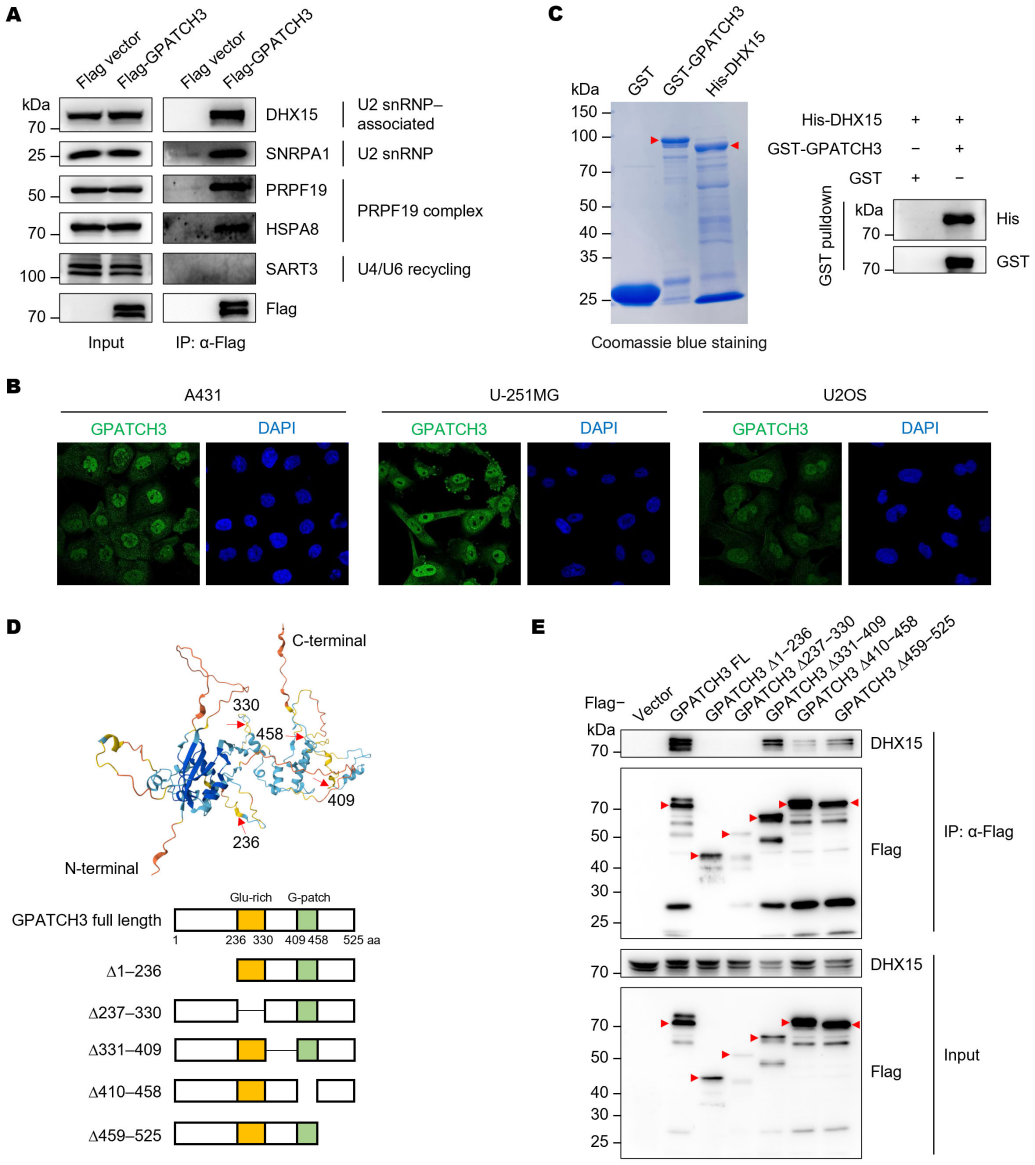
GPATCH3 directly interacts with the RNA helicase DHX15. **(A)** Flag or Flag-tagged GPATCH3 was expressed in HEK293T cells and subjected to immunoprecipitation using anti-Flag M2 beads. Immunoblotting was performed with the indicated antibodies. **(B)** Subcellular localization of GPATCH3 in various human cells, as reported by the Human Protein Atlas. **(C)** Purified GST or GST-tagged GPATCH3 proteins were incubated with His-tagged DHX15, followed by GST pulldown and immunoblotting for His and GST. **(D)** Schematic of GPATCH3 truncation mutants based on AlphaFold-predicted structural domains. **(E)** HEK293T cells were transfected with Flag vector, Flag-tagged full-length GPATCH3, or GPATCH3 truncation mutants. Cell lysates were immunoprecipitated with anti-Flag M2 beads and analyzed by immunoblotting with the indicated antibodies.

To validate the Co-IP/MS findings, we immunoprecipitated Flag-tagged GPATCH3 and detected the co-precipitated proteins via western blotting (**Figure 2A**). The results confirmed that GPATCH3 interacts with U2 snRNP and associated proteins, such as DHX15 and SNRPA1, and the PRPF19 complex, including PRPF19 and HSPA8 (**Figure 2A**). Immunofluorescence-based subcellular localization analysis in multiple cell lines showed that GPATCH3 is predominantly distributed in the nucleus (**Figure 2B**), further supporting its potential involvement in splicing regulation.

We next sought to determine whether GPATCH3 directly interacts with its binding partner. GST pulldown assays confirmed a direct interaction between GPATCH3 and DHX15 (**Figure 2C**). To map the structural domains responsible for this interaction, we generated a series of GPATCH3 truncation mutants guided by structural predictions from AlphaFold (**Figure 2D**). Deletion of either the N-terminal region (amino acids 1–236) or the G-patch domain (amino acids 410–458) abrogated the interaction with DHX15 (**Figure 2E**), indicating that both regions are required for their association.

Taken together, these findings suggest that GPATCH3 may participate in pre-mRNA splicing through its physical and functional interaction with DHX15.

### 2.3 GPATCH3 enhances DHX15 ATPase activity and modulates alternative splicing

**Figure 3:**
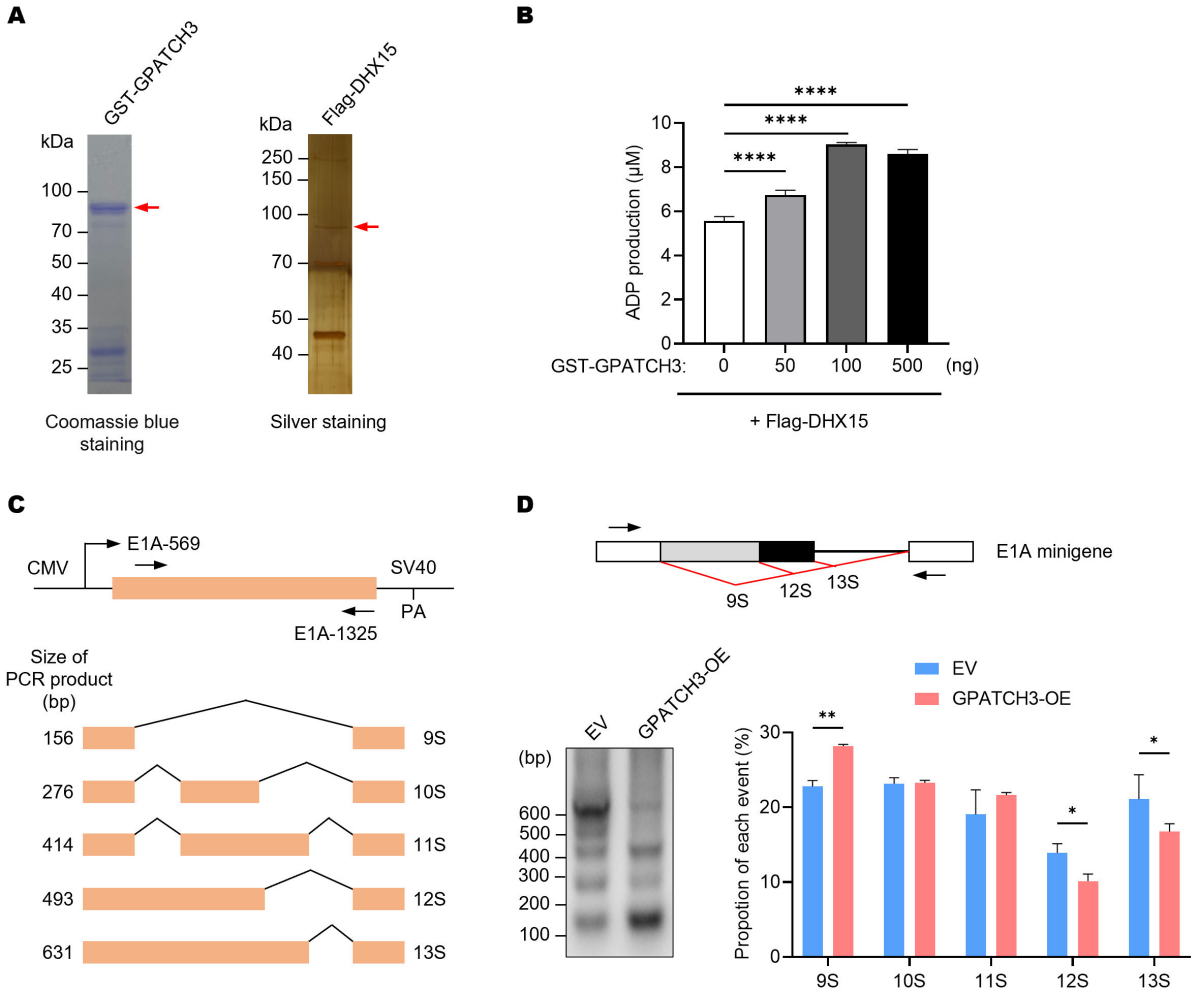
GPATCH3 enhances the ATPase activity of DHX15 and modulates alternative splicing. **(A)** Purification of GST-tagged GPATCH3 from *E coli* and Flag-tagged DHX15 from transfected HEK293T cells. Purified proteins were assessed by Coomassie blue and silver staining. **(B)** GPATCH3 (increasing amounts) was co-incubated with 100 ng DHX15 in the presence of 100 μM ATP for 30 min at 37°C. ADP production was measured to evaluate DHX15 ATPase activity. Data are presented as mean ± SD. *P* values were calculated using one-way ANOVA with Dunnett’s multiple comparisons test. ****P < 0.0001. **(C)** Diagram of the E1A minigene (pCMV-E1A) illustrating alternative splicing events that produce the 13S, 12S, 11S, 10S, and 9S transcript isoforms. Positions of primers used for RT-PCR are shown. **(D)** RT-PCR analysis of E1A minigene transcripts in HEK293T cells co-transfected with pCMV-E1A along with control vector or GPATCH3 overexpression plasmid. Representative gel image (left) and quantification of isoform distribution (right) are shown. Data are presented as mean ± SD. *P* values were determined by two-way ANOVA. *P < 0.05; **P < 0.01.

Previous studies have shown that certain G-patch proteins, such as GPATCH2, GPATCH4, and RBM5, can directly bind to DHX15 via their G-patch domains and markedly enhance its ATPase activity, thereby contributing to spliceosome disassembly and intron lariat resolution (22, 30, 31). We therefore investigated whether GPATCH3 exerts a similar regulatory effect. *In vitro* ATPase assays revealed that GPATCH3 significantly stimulated the ATPase activity of DHX15 in a dose-dependent manner (**Figures 3A, B**). These results indicate that GPATCH3 can function as a cofactor to potentiate DHX15 enzymatic activity.

Given that DHX15 activity governs the dynamic remodeling of the spliceosome and affects pre-mRNA splicing fidelity, we next assessed whether GPATCH3 influences alternative splicing outcomes. Using an E1A minigene reporter system (32), we showed that GPATCH3 overexpression reduced usage of the most proximal 5′ splice site, resulting in decreased levels of the 12S/13S isoforms and increased levels of the 9S isoform (**Figures 3C, D**). These data demonstrate that GPATCH3 modulates pre-mRNA alternative splicing.

### 2.4 Elevated GPATCH3 expression correlates with poor prognosis in human cancers and in mouse xenografts

**Figure 4:**
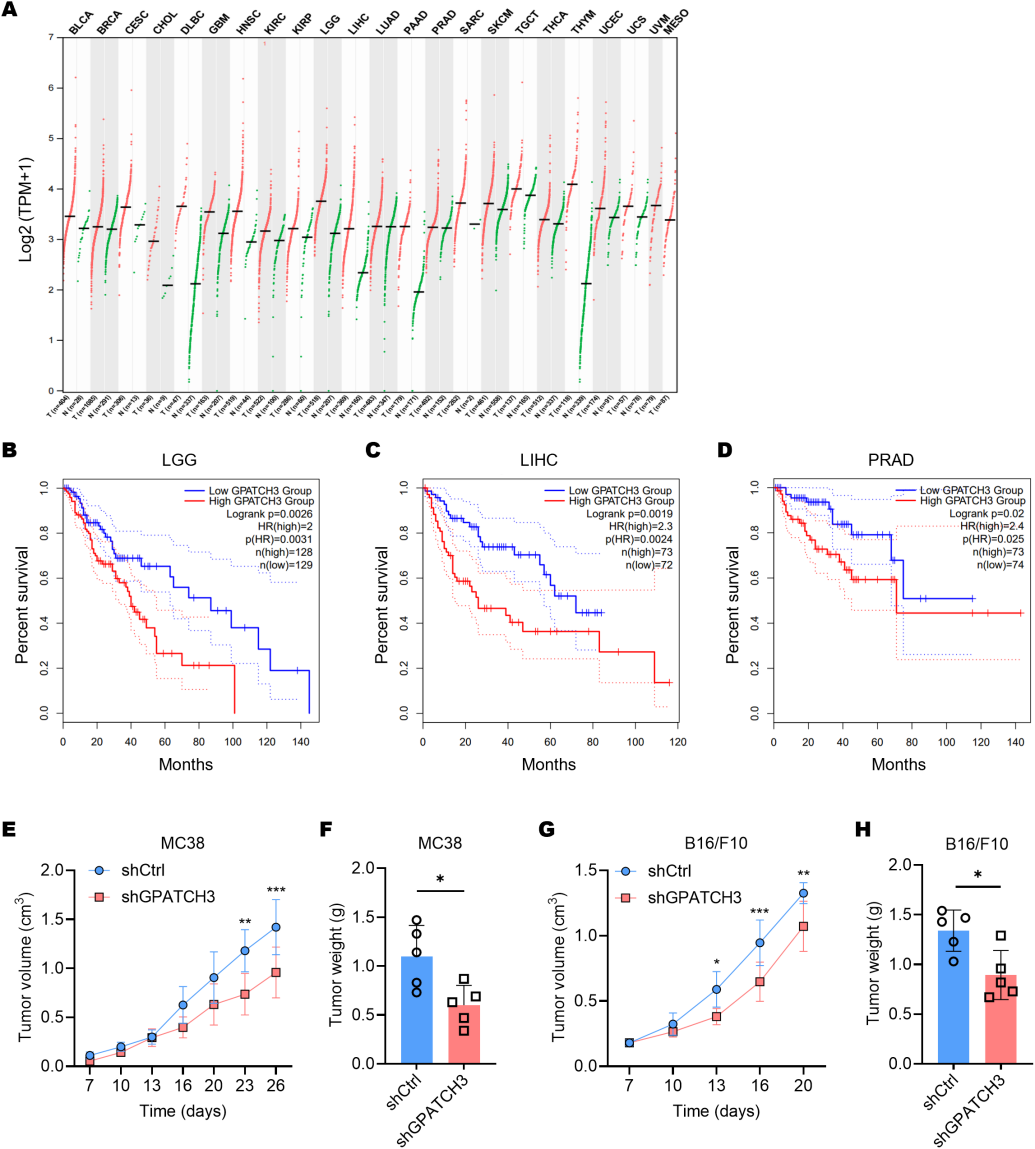
Elevated GPATCH3 expression is associated with poor prognosis in human cancers and in mouse xenografts. **(A)** Dot plot showing GPATCH3 expression across tumor and paired normal tissues from TCGA and GTEx datasets. Each dot represents one sample; horizontal lines indicate median values. UVM and MESO lack normal controls. TPM, transcripts per million. **(B–D)** Kaplan–Meier survival analyses showing the correlation between GPATCH3 expression and overall survival in patients with LGG (lower-grade glioma; **(B)**, LIHC (liver hepatocellular carcinoma; **(C)**, and PRAD (prostate adenocarcinoma; D), plotted by GEPIA2. **(E, F)** Tumor volumes **(E)** and tumor weights **(F)** in C57BL/6 mice subcutaneously injected with control or GPATCH3-depleted MC38 cells (n = 5 mice per group). **(G, H)** Tumor volumes **(G)** and tumor weights H in C57BL/6 mice subcutaneously injected with control or GPATCH3-depleted B16/F10 cells (n = 5 mice per group). Data are presented as mean ± SD. *P* values were calculated by two-way ANOVA **(E, G)** and unpaired two-tailed Student’s *t*-test **(F, H)**. *P < 0.05; **P < 0.01; ***P < 0.001.

Pan-cancer transcriptomic analysis using GEPIA2 (33) revealed that GPATCH3 is significantly upregulated in a broad spectrum of malignancies compared to corresponding normal tissues (**Figure 4A**). To explore the clinical relevance of this upregulation, we performed survival analyses using TCGA datasets. Kaplan–Meier curves demonstrated that high GPATCH3 expression is associated with reduced overall survival in multiple cancer types, including lower-grade glioma (LGG), liver hepatocellular carcinoma (LIHC), and prostate adenocarcinoma (PRAD) (**Figures 4B–D**).

Consistent with these findings, *in vivo* functional studies showed that depletion of GPATCH3 significantly suppressed tumor growth in mouse xenograft models (**Figures 4E–H**). Together, these data highlight GPATCH3 as a potential oncogenic factor that promotes tumor progression and may serve as a prognostic biomarker in certain cancers.

### 2.5 GPATCH3 is linked to an immunosuppressive tumor microenvironment

**Figure 5:**
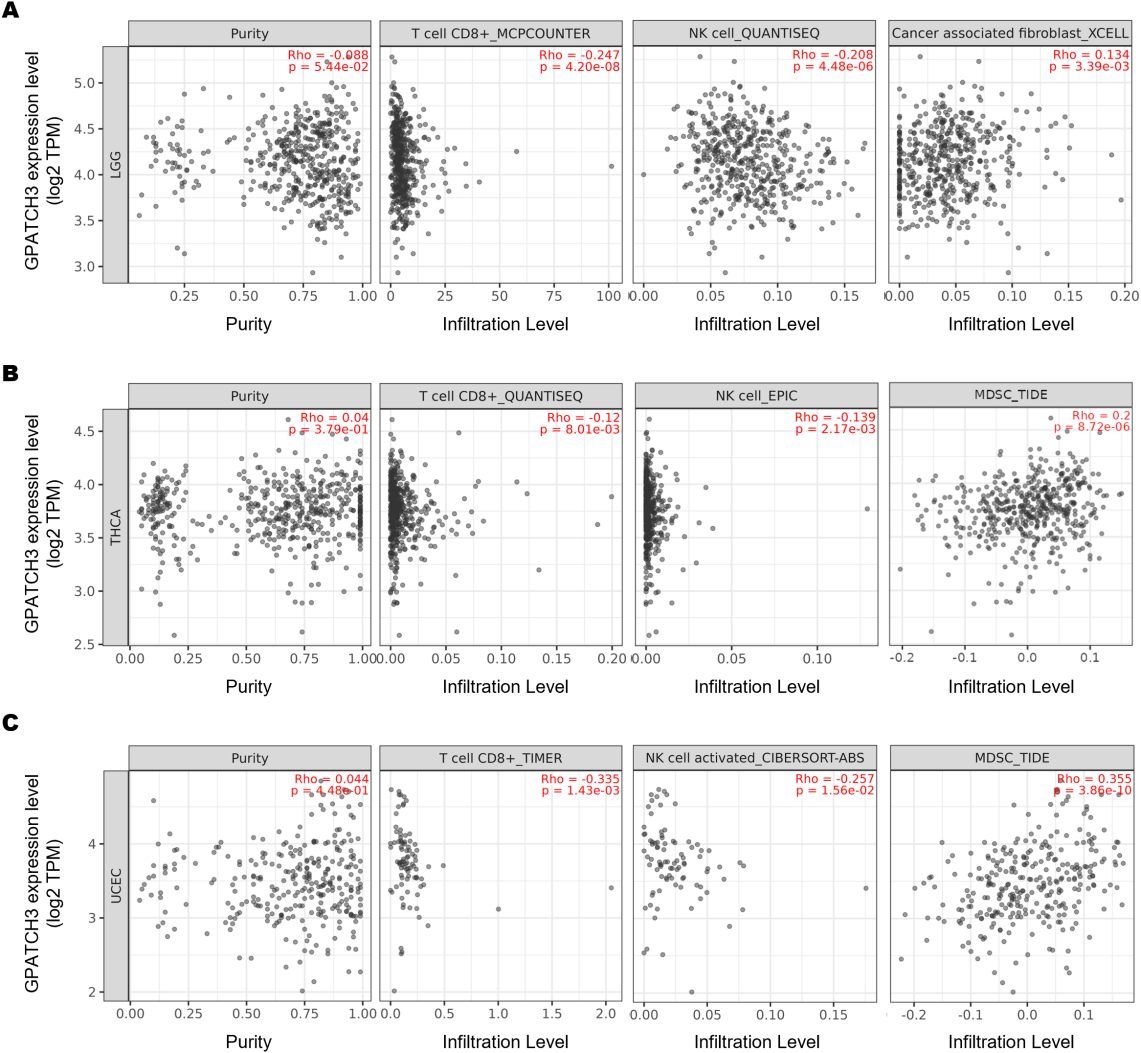
GPATCH3 expression correlates with immune cell infiltration in the TME. **(A−C)** Correlations between GPATCH3 expression and infiltration of CD8^+^ T cells, NK cells, cancer-associated fibroblasts (CAFs), and myeloid-derived suppressor cells (MDSCs) across different cancers. Data were derived from TIMER2.0, adjusted for tumor purity. Representative examples from LGG (lower-grade glioma; **(A)**, THCA (thyroid carcinoma; **(B)**, and UCEC (uterine corpus endometrial carcinoma; **(C)** are shown.

The dynamic interplay between tumors and their surrounding stromal and immune constituents—collectively termed the tumor microenvironment (TME)—has emerged as a critical determinant of cancer progression and therapy response (34–36). Given the well-established role of immune cell infiltration in modulating antitumor immunity, we hypothesized that GPATCH3, a poorly characterized gene in cancer biology, might influence TME composition. To test this, we employed TIMER2.0 (37) to assess the immunomodulatory impact of GPATCH3. Notably, GPATCH3 expression was significantly negatively correlated with cytotoxic CD8^+^ T cells and NK cell infiltration, while showing positive correlations with immunosuppressive populations such as myeloid-derived suppressor cells (MDSCs) and cancer-associated fibroblasts (CAFs) across multiple cancer types (**Figures 5A–C**).

**Figure 6:**
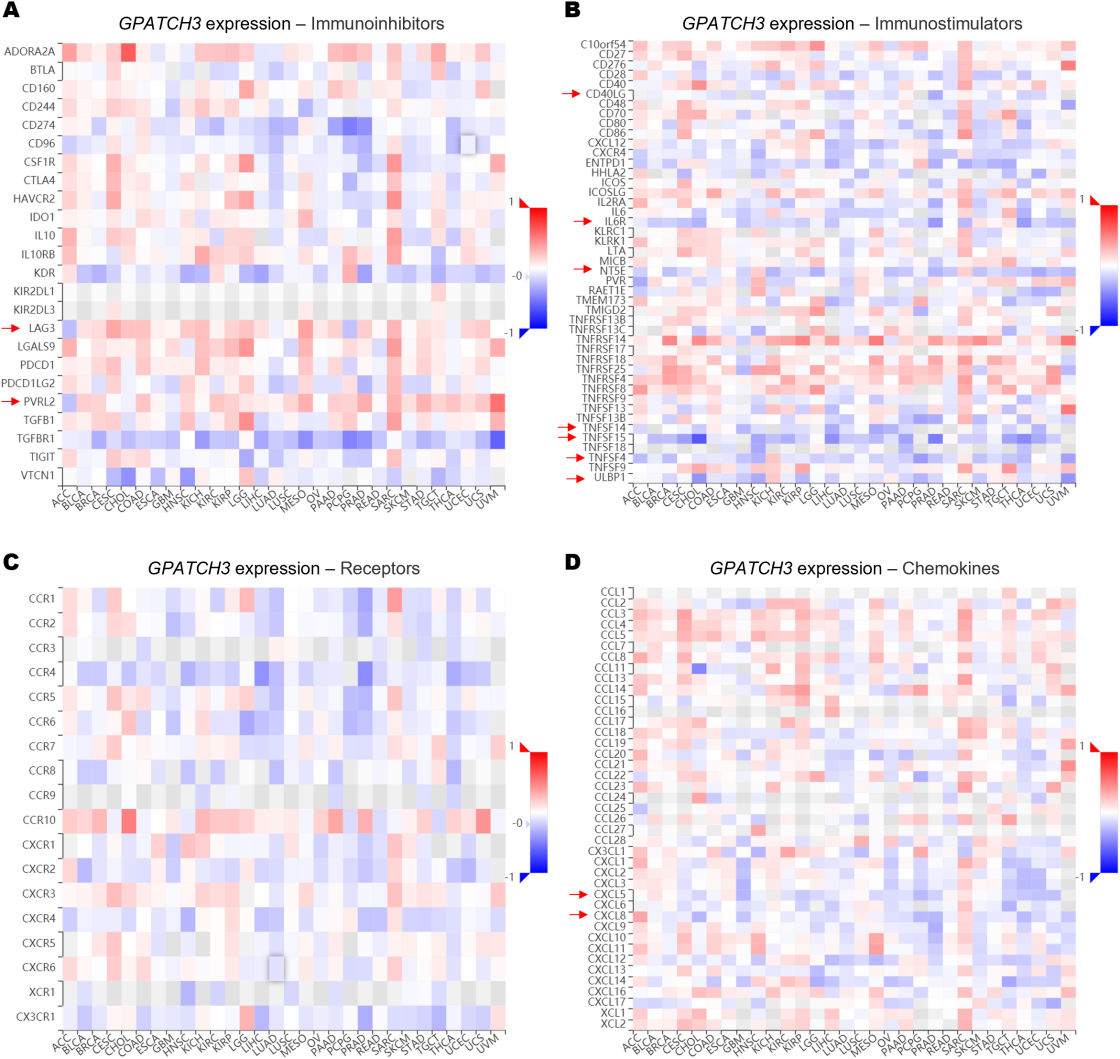
GPATCH3 expression is associated with immune regulatory gene signatures. **(A−D)** Heatmaps showing the correlations between GPATCH3 expression and the abundance of immunoinhibitory molecules **(A)**, immunostimulatory molecules **(B)**, immune receptors **(C)**, and chemokines **(D)**. The data are plotted by TIMER2.0. Regulatory genes with consistent trends across cancer types are indicated with arrows. .

Since the extent of immune cell infiltration is closely influenced by the dynamic balance of immunoregulatory factors, we next evaluated the associations between GPATCH3 and key classes of immune modulators, including immunoinhibitors, immunostimulators, receptors, and chemokines. Heatmap analyses revealed that GPATCH3 expression was broadly negatively correlated with chemokines such as CXCL5 and CXCL8, as well as immunostimulatory molecules including CD40LG and TNFSF15, in a pan-cancer context (**Figures 6A–D**). These alterations may contribute to the reprogramming of immune infiltration patterns within the TME.

Together, these findings from multiple lines of analysis support a model in which GPATCH3 functions as a key orchestrator of the immunosuppressive TME, likely through regulating the splicing and expression of immune-related genes involved in these pathways.

### 2.6 GPATCH3 deficiency induces splicing abnormalities and alters the expression of immunoregulatory genes

**Figure 7:**
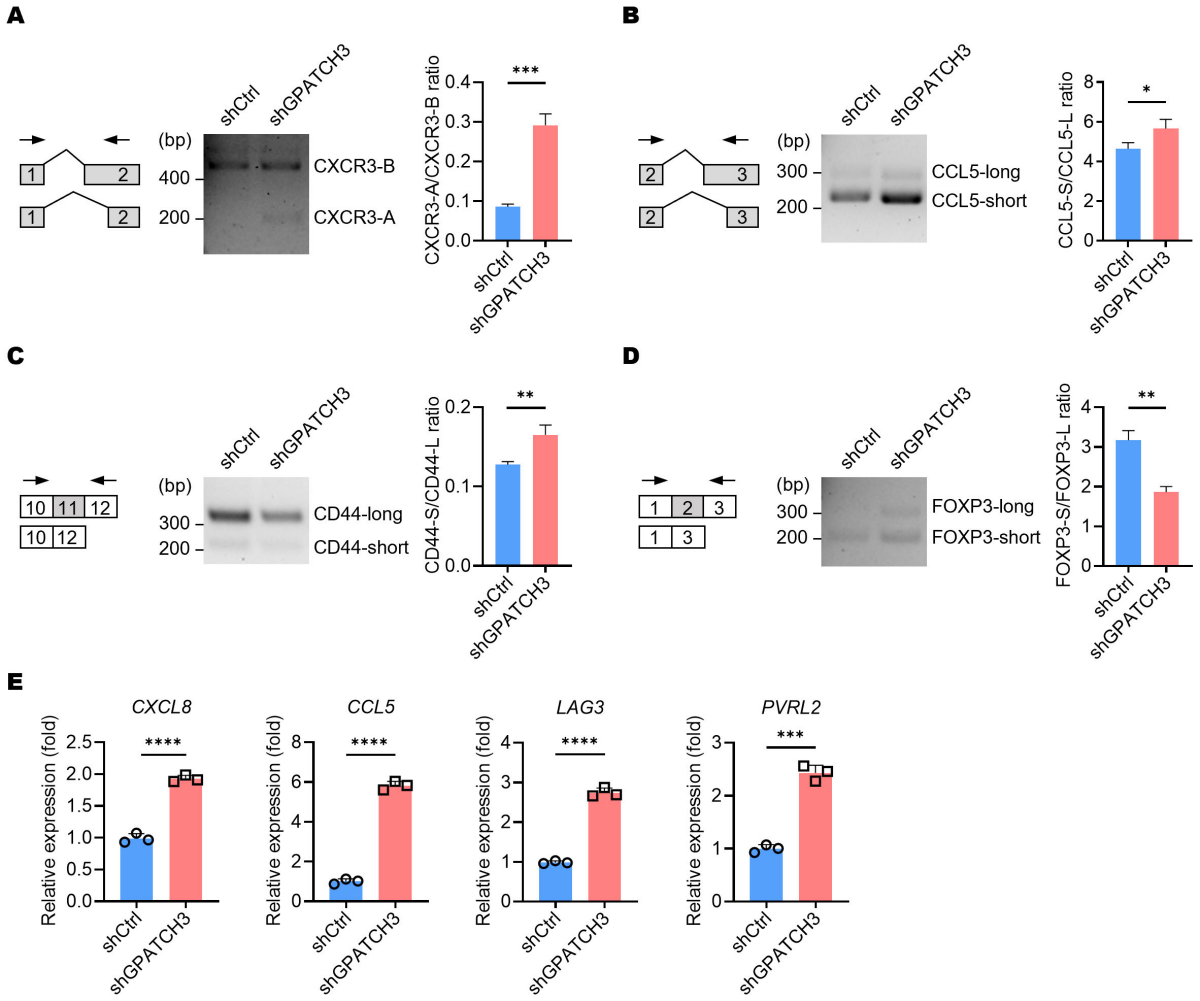
GPATCH3 deficiency alters alternative splicing and expression of immune regulatory genes. **(A–D)** A549 cells were transduced with control (shCtrl) or GPATCH3-targeting shRNA (shGPATCH3). Seventy-two hours post-infection, total RNA was extracted and subjected to RT-PCR using isoform-specific primers (indicated arrows). Representative fragment analysis of splicing changes is shown (CXCR3, **(A)**; CCL5, **(B)**; CD44, **(C)**; FOXP3, **(D, E)** RT-qPCR analysis of immune regulatory genes in control versus GPATCH3-depleted A549 cells. Data are presented as mean ± SD. *P* values were determined by unpaired two-tailed Student’s *t*-test. *P < 0.05; **P < 0.01; ***P < 0.001; ****P < 0.0001.

Given the role of GPATCH3 in alternative splicing regulation, we investigated whether its deficiency alters the splicing patterns of key immunoregulatory genes known to generate functionally distinct isoforms. For example, *CXCR3* produces two major isoforms—*CXCR3-A* and *CXCR3-B*—through alternative 3′ splice site selection within exon 2 (38). *CXCR3-B* utilizes a more proximal 3′ splice site, resulting in a longer extracellular N-terminus compared to *CXCR3-A*. Functionally, CXCR3-A promotes cell proliferation, survival, and chemotaxis, whereas CXCR3-B is associated with growth inhibition and apoptosis (39). We observed an increased ratio of the *CXCR3-A* isoform upon GPATCH3 depletion (**Figure 7A**).

Similarly, alternative splicing of *CCL5* was also perturbed in the absence of GPATCH3 (**Figure 7B**). Given that non-canonical *CCL5* isoforms may influence mRNA stability or translational efficiency, these alterations suggest a potential mechanism by which GPATCH3 modulates chemokine signaling. We further identified aberrant splicing events in additional immune-related genes, including *CD44* and *FOXP3* (**Figures 7C, D**). Together, these results suggest that GPATCH3 modulates alternative splicing of immuneregulatory genes, thereby may affect immune infiltration.

Because alternative splicing can influence gene expression levels either directly or indirectly, we further investigated the impact of GPATCH3 deficiency on the gene expression. Notably, depletion of GPATCH3 led to significant upregulation of *CXCL8*, *CCL5*, *LAG3*, and *PVRL2* (**Figure 7E**)—genes with well-established roles in immune cell recruitment, immune checkpoint signaling, and tumor–immune interactions.

Collectively, our findings demonstrate that loss of GPATCH3 disrupts both the splicing and expression of genes involved in immune modulation, thereby potentially reshaping the immune landscape of the TME.

## 3 Discussion

In this study, we identified GPATCH3 as a novel splicing regulator that interacts with DHX15, a DEAH-box helicase critical for spliceosome remodeling. This interaction is mediated through both the N-terminal and conserved G-patch domains of GPATCH3. Functionally, GPATCH3 enhances the ATPase activity of DHX15, suggesting that it may act as a cofactor to fine-tune the enzymatic steps of spliceosomal transitions. Given that DHX15 plays a crucial role in late-stage spliceosome disassembly and lariat intron turnover, GPATCH3 may be particularly important for ensuring proper spliceosome recycling and maintaining the fidelity of alternative splicing. Indeed, using the E1A minigene system (32), we further demonstrated that GPATCH3 modulates alternative splice site selection. Overexpression of GPATCH3 shifted the balance among E1A isoforms, suggesting a role in regulating splicing dynamics. These observations align with reports on other G-patch proteins, such as GPATCH1 and GPATCH4, which similarly regulate splicing fidelity through interactions with RNA helicases (21, 30).

Spliceosome dysregulation has emerged as a key factor in cancer development. Aberrant splicing of downstream targets may disrupt cellular homeostasis, facilitate immune evasion, and contribute to oncogenesis (5, 6, 40, 41). Our pan-cancer analyses revealed that GPATCH3 is upregulated in various tumor types and is significantly correlated with poor patient survival in glioma, liver, and prostate cancers. In functional assays, GPATCH3 depletion suppressed tumor growth in xenograft models, supporting its pro-tumorigenic role.

Importantly, our study uncovers a previously unrecognized immunomodulatory function of GPATCH3. Transcriptomic and splicing analyses revealed that GPATCH3 deficiency perturbs the splicing patterns of key immunoregulatory genes—including *CXCR3*, *CCL5*, *CD44*, and *FOXP3*—all of which have isoforms with distinct, and sometimes opposing, immune functions. For instance, we observed a shift toward the *CXCR3-A* isoform, which promotes chemotaxis, upon GPATCH3 depletion. Moreover, we found significant upregulation of immune checkpoint and chemokine genes such as *CXCL8*, *LAG3*, and *PVRL2*, suggesting that GPATCH3 loss affects isoform diversity and expression of functional isoforms.

Mechanistically, one plausible explanation is that GPATCH3 deficiency leads to widespread production of aberrant transcripts harboring premature stop codons or exon skipping events, which may be targeted by nonsense-mediated mRNA decay (NMD) pathways (42), thereby indirectly influencing gene expression networks that regulate immune infiltration and activation states. This hypothesis aligns with our TIMER2.0-based analyses, which revealed that GPATCH3 expression negatively correlates with cytotoxic immune populations, such as CD8^+^ T cells and NK cells, and positively associates with immunosuppressive cells like MDSCs and CAFs. Thus, GPATCH3 appears to contribute to the establishment and maintenance of an immunosuppressive TME, potentially through splicing-dependent regulation of immune signaling components.

These findings position GPATCH3 as a critical node linking spliceosome dynamics to tumor immunity. This connection is particularly relevant in the context of emerging cancer therapies targeting the splicing machinery. Several small molecules, such as E7107 and H3B-8800, are currently under investigation for their ability to selectively disrupt splicing in cancer cells (15–17, 43, 44). Our results suggest that targeting GPATCH3, or its functional interaction with DHX15, could offer an alternative strategy to perturb oncogenic splicing programs while simultaneously reversing immune evasion phenotypes.

Furthermore, combining GPATCH3-targeted interventions with immune checkpoint blockade may synergistically enhance anti-tumor immunity. For instance, splicing modulation might increase the generation of neoantigens or restore the expression of stimulatory chemokines, thereby enhancing the efficacy of immunotherapy. Since immunosuppressive microenvironments often underlie resistance to checkpoint inhibitors (45), targeting GPATCH3 could potentially reprogram the immune landscape in favor of tumor clearance.

Nevertheless, our study has several limitations. While we provide compelling evidence for the role of GPATCH3 in alternative splicing and immune regulation, the precise downstream splicing events that mediate these phenotypes remain incompletely defined. High-resolution RNA-binding and crosslinking assays (e.g., CLIP-seq) would help delineate direct RNA targets of the GPATCH3–DHX15 complex. In addition, the tissue-specific roles of GPATCH3 in different tumor contexts, as well as its potential non-splicing functions, warrant further investigation. Future studies should also explore how GPATCH3 expression is regulated—whether through epigenetic mechanisms, oncogenic signaling, or microRNA-mediated repression. Understanding these upstream controls may reveal new vulnerabilities in cancers with high GPATCH3 activity. Moreover, *in vivo* studies using different immunocompetent models would be valuable to elucidate how GPATCH3-driven splicing reprogramming affects immune cell composition and tumor–immune dynamics in a physiologically relevant setting.

In conclusion, our study establishes GPATCH3 as a previously unrecognized splicing regulator that mechanistically links spliceosome activity to tumor progression and immune modulation. Through its interaction with DHX15, GPATCH3 safeguards alternative splicing fidelity and regulates the expression of immunoregulatory genes. These findings not only expand the repertoire of cancer-associated splicing factors but also highlight novel molecular vulnerabilities for spliceosome-targeted interventions.

## 4 Materials and methods

### 4.1 Antibodies

Primary antibodies used for immunoblotting included: anti-PRPF19 (Cat. # A12590), anti-SART3 (Cat. # A12124), anti-SNRPA (Cat. # A6410), anti-HSPA8 (Cat. # A2487) from ABclonal; anti-DHX15 (Cat. # L2221) from Santa Cruz Biotechnology; anti-FLAG (clone M2, Cat. # F1804), anti-GST (Cat. # G1160) from Sigma-Aldrich; anti-His (clone 27E8, Cat. # 2366) from Cell Signaling Technology. Secondary antibodies were Peroxidase-Conjugated AffiniPure Goat Anti-Rabbit IgG (H+L) (Cat. # 111-035-003; Jackson Lab) and Goat Anti-Mouse IgG (H+L) (Cat. # 115-035-003; Jackson Lab).

### 4.2 Cell culture and authentication

A549, HEK293T, MC38, and B16/F10 cell lines were obtained from the American Type Culture Collection (ATCC) and maintained in Dulbecco’s modified Eagle’s medium (DMEM; Gibco) supplemented with 10% heat-inactivated fetal bovine serum (Lonsera), 100 U/mL of penicillin and 100 µg/mL of streptomycin at 37°C in a humidified incubator with 5% CO_2_. Prior to experimentation, cell line authenticity was verified by short tandem repeat (STR) profiling, and routine mycoplasma testing using PCR-based detection confirmed the absence of mycoplasma contamination throughout the study period.

### 4.3 Mouse xenograft models

Six- to eight-week-old C57BL/6 mice were randomly assigned to two groups (5 mice per group) and injected subcutaneously with either GPATCH3-depleted MC38 or B16/F10 tumor cells, or corresponding control cells. At the experimental endpoint, mice were anesthetized and euthanized. Tumors were excised and measured for volume and weight.

### 4.4 Plasmids and shRNA constructs

Flag-tagged GPATCH3 plasmid was generated by cloning the human *GPATCH3* coding sequence (CDS) into pcDNA6-N-Flag vectors. Plasmids expressing GST-tagged and His-tagged proteins were generated by inserting the CDS into pGEX-4T-1 and pET-28a (+) vectors, respectively. Lentiviral knockdown plasmids were constructed by inserting the annealed shRNAs into pLV-H1-EF1α-puro vector (Cat. # SORT-B19; Biosettia). The shRNA sequence specifically targeting GPATCH3 was: 5′–GGACATGAGTGTGTACTATGA–3′ (shGPATCH3). The nontargeting control shRNA (shCtrl) sequence was 5′–CAACAAGATGAAGAGCACC–3′. All constructs were validated by Sanger sequencing.

### 4.5 Lentivirus production and stable cell line generation

Lentiviral particles were produced in HEK293T cells by co-transfecting the lentiviral vector with three packaging plasmids (pVSV-G, pMDLg/pRRE, and pRSV-Rev) using calcium phosphate precipitation. Target cells were transduced with the resulting viral supernatant by spinoculation (1,000× g, 30 min at 32°C) in the presence of 8 μg/mL polybrene (Cat. # H9268; Sigma-Aldrich). Stable cell lines were generated by selecting transduced cells with puromycin (1–2 μg/mL) starting 48 hours post-transduction.

### 4.6 Immunoprecipitation and mass spectrometry analysis

Cells were washed with ice-cold phosphate-buffered saline (PBS) and lysed with lysis buffer (25 mM Tris-HCl, pH 7.4; 150 mM NaCl; 1% NP-40; 1 mM EDTA; 1 mM EGTA; 1× protease and phosphatase inhibitor cocktail). Samples were incubated on ice for 30 min and sonicated for 15 seconds. Cell lysates were centrifuged at 12,000 × g for 15 min at 4°C. Ten percent of the supernatant was prepared as the input. For immunoprecipitation of Flag-tagged proteins, the supernatant was incubated with anti-Flag M2 magnetic beads (Cat. # M8823; Sigma-Aldrich) at 4°C for 4 h on a rotor. After immunoprecipitation, the beads were washed four times with lysis buffer. Then, 3× Flag peptide (Cat. # F4799; Sigma-Aldrich) was added to elute the bound proteins from beads. Proteins in the supernatants were resolved on SDS–PAGE and detected by immunoblotting or silver staining (Cat. # C510027; Sangon Biotech). To identify GPATCH3-interacting partners, the eluted proteins were subjected to silver staining and semi-quantitative mass spectrometry (liquid chromatography-tandem mass spectrometry) analysis by the Proteomics Core Facility at the Institutes of Biomedical Sciences, Fudan University.

### 4.7 Immunoblotting

Proteins were quantified by BCA assay (Cat. # ZJ102; Epizyme), separated by SDS–polyacrylamide gel electrophoresis (SDS–PAGE) and transferred to Nitrocellulose membranes (Cat. # 10600002; Cytiva). The membranes were blocked with 5% nonfat milk in Tris-buffered saline at room temperature for 1 h and then probed with primary antibodies overnight at 4°C, followed by HRP-conjugated secondary antibodies at room temperature for 1 h. Protein signals were finally detected using an enhanced chemiluminescence (ECL) system (Cat. # SB-WB012; Share-Bio) and captured with a digital imaging system (Tanon).

### 4.8 GST pulldown assay

GST- or His-tagged proteins were expressed in BL21 (DE3) bacteria and grown in LB media until the OD600 reaches 0.8~1. Then, the cultures were induced at 18°C overnight with 0.2 mM isopropyl β-D-thiogalactopyranoside (IPTG; Cat. # A100487; Sangon Biotech). Bacterial extracts were prepared by sonication in PBS supplemented with protease inhibitors, followed by centrifugation at 12,000 rpm at 4°C for 15 min. The supernatants were incubated with glutathione agarose beads (Cat. # 16100; Thermo Fisher) on a rotating mixer for 2 h at 4°C. The beads were washed three times and the purified proteins were ready for GST pulldown assay. His-tagged proteins were purified similarly using Ni-NTA Magarose Beads (Cat. # SM008025; Smart-Lifesciences) and eluted with imidazole (Cat. # A600277; Sangon Biotech). His-tagged proteins were incubated with GST or GST-tagged GPATCH3 proteins bounded to glutathione agarose beads in binding buffer (0.1% NP-40 in PBS). The binding reaction was performed 2 h with a rotating mixer at 4°C, after which the beads were washed three times. GST proteins were eluted with elution buffer (50 mM Tris-HCl, pH 8.0, 150 mM NaCl, 10 mM reduced glutathione). The samples were boiled at 95°C for 5 min and subjected to Western blot analysis.

### 4.9 ATPase activity assay

GST-GPATCH3 proteins were purified from *E.coli* and Flag-tagged DHX15 proteins were purified from transfected HEK293T cells. Different doses of GPATCH3 were incubated at 37°C with 100 ng DHX15 for 30 min in the reaction buffer (50 mM HEPES, pH 7.4, 150 mM NaCl, 5% Glycerol, 10 mM MgCl_2,_ 0.1% NP-40, 1 mM DTT). Following incubation, ATP was added at a final concentration of 100 μM and incubated for another 30 min at 37°C. Then the reaction was detected by Malachite Green Phosphate Detection Kit (Cat. # S0196M; Beyotime) according to the manufacturer’s protocol.

### 4.10 Reverse transcription PCR

Total RNA was extracted using TRIzol reagent (Cat. # R401-01; Vazyme) and reverse transcribed into cDNA using HiScript III RT SuperMix (Cat. # R323-01; Vazyme). Semi-quantitative RT–PCR and a Tanon Bio-Fragment Analyzer (BiOptic) were used to analyze alternative spliced products. The oligonucleotides used for RT–PCR were as follows: ElA569, 5′–ATTATCTGCCACGGAGGTGT–3′; ElA1315, 5′–GGATAGCAGGCGCCATTTTA–3′; CXCR3-RT-F, 5′–CCCAGCAGCCAGAGCACCAGC–3′; CXCR3-RT-R, 5′–CAGGAAGGCCCGGTCGAAGTTC–3′; CCL5-RT-F, 5′–CACCCTGCTGCTTTGCCTACA–3′; CCL5-RT-R, 5′–AGGTTCAAGGACTCTCCATCC–3′; CD44-RT-F, 5′–GGAAGAAACAGCTACCCAGA–3′; CD44-RT-R, 5′–CATTGAAAGAGGTCCTGTCC–3′; FOXP3-RT-F, 5′–TCCAGGGCCGAGATCTTCGAG–3′; FOXP3-RT-R, 5′–TGAGGGAGAAGACCCCAGTGG–3′.

The oligonucleotides used for RT–qPCR were as follows: CXCL8-RT-F, 5′–ATACTCCAAACCTTTCCACCC–3′; CXCL8-RT-R, 5′–TCTGCACCCAGTTTTCCTTG–3′; LAG3-RT-F, 5′–CCTACACCTGCCATATCCATC–3′; LAG3-RT-R, 5′–AGCGTTCTTGTCCAGATACTG–3′; CCL5-RT-F, 5′–TGCCCACATCAAGGAGTATTTC–3′; CCL5-RT-R, 5′–CCATCCTAGCTCATCTCCAAAG–3′; PVRL2-RT-F, 5′–GCATGAGAGCTTCGAGGAAC–3′; PVRL2-RT-R, 5′–GGAGATGGACACTTCAGGAG–3′.

### 4.11 Statistical analysis

Data in the study are presented as means ± S.D. and analyzed statistically using GraphPad Prism 8.0 software. *P* values were calculated using unpaired two-tailed Student’s *t*-test between two groups and one-way or two-way ANOVA for multiple groups, respectively. *P* < 0.05 was considered statistically significant.

## Data Availability

The original contributions presented in the study are included in the article/**Supplementary Material**. Further inquiries can be directed to the corresponding author.
