# Supplementary figures and images for: GPATCH3, a splicing regulator that facilitates tumor immune evasion via the modulation of ATPase activity of DHX15
Source: Front Immunol. 2025 Aug 11;16:1612461. doi: 10.3389/fimmu.2025.1612461 (PMC12375585; doi:10.3389/fimmu.2025.1612461)

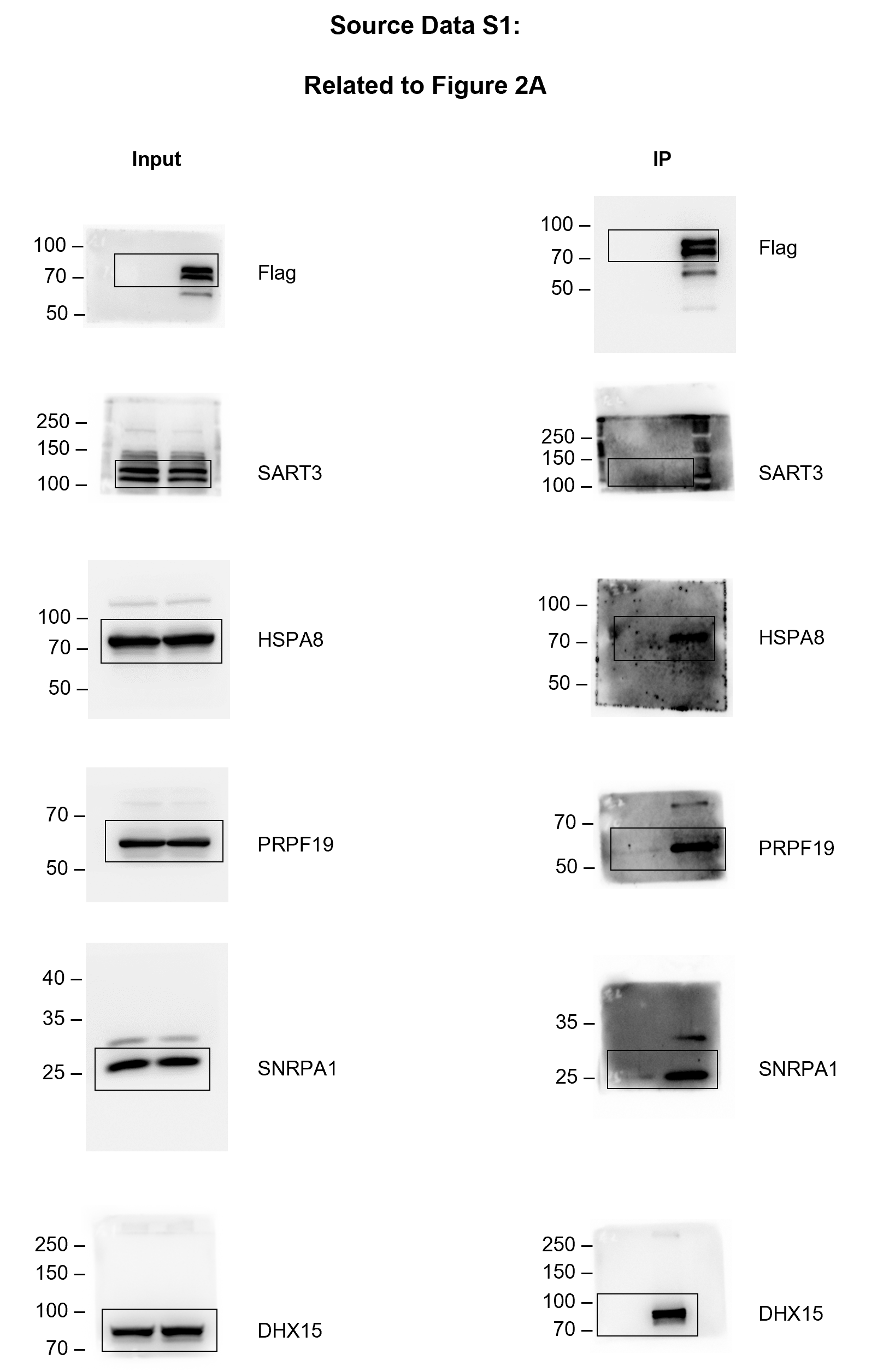

Supplement: Supplementary file 2 [file Image1.tif]

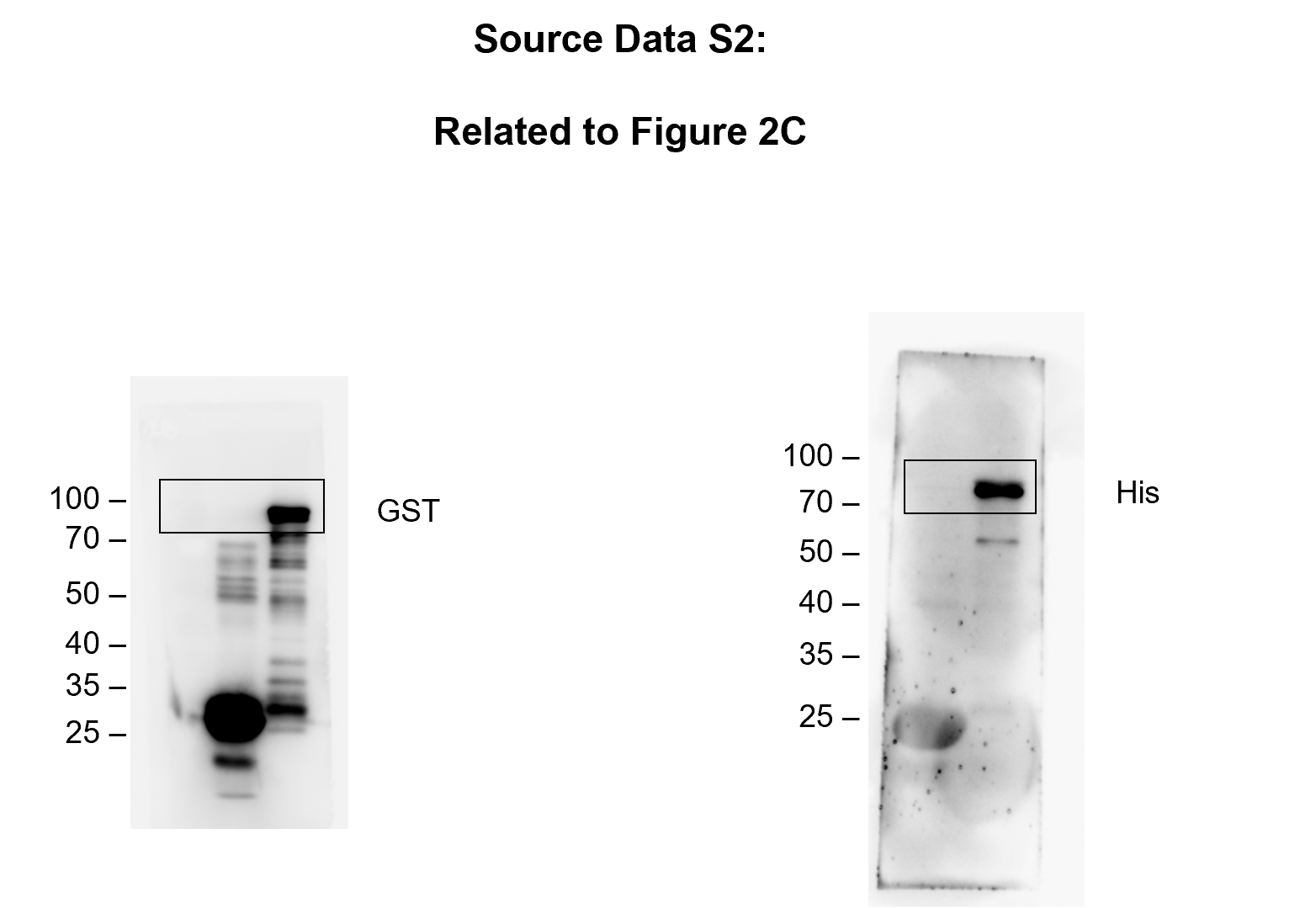

Supplement: Supplementary file 3 [file Image2.tif]

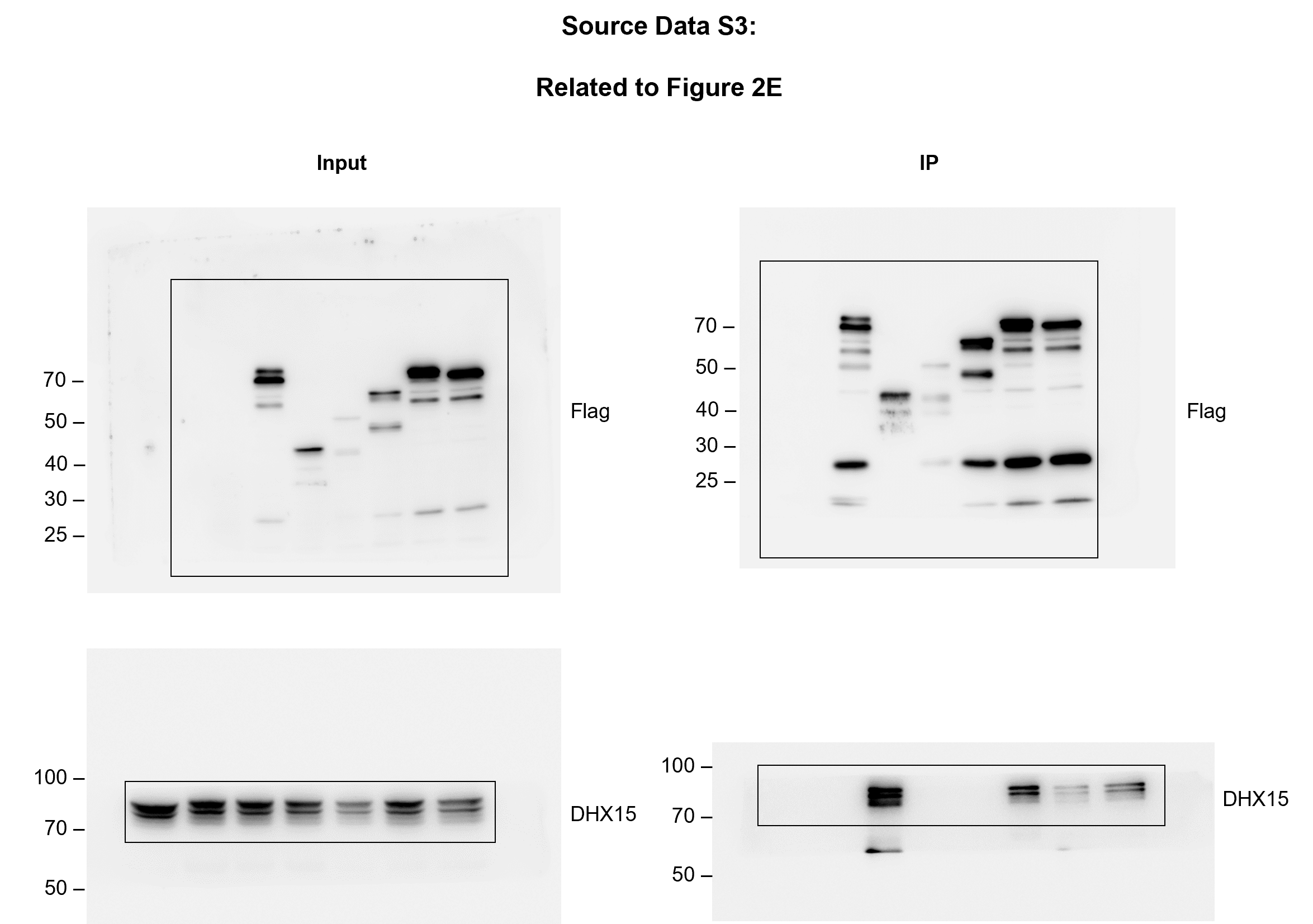

Supplement: Supplementary file 4 [file Image3.tif]
